# EARLY LIFE LEARNING DISABILITY, PREMATURE PARENTAL DEATH PREDICTS LATE LIFE COGNITIVE IMPAIRMENT

**DOI:** 10.1093/geroni/igad104.3292

**Published:** 2023-12-21

**Authors:** Allan Nkwata, Jacqui Smith

**Affiliations:** University of Michigan, Ann Arbor, Michigan, United States; University of Michigan, Ann Arbor, Michigan, United States

## Abstract

Recent studies have increasingly identified links between early learning difficulties and later life cognitive impairment. Additionally, the death of a parent adversely affects various outcomes across the life course, particularly when the death occurs earlier than expected. This study evaluated the impact of early learning problems and death of a parent on change in cognitive function over 10 years among older Americans enrolled in the Health and Retirement Study. Data were from respondents’ early educational experiences collected in the 2015 and 2017 Life History Mail Survey and childhood stress measures were from the aggregated childhood histories (N = 10,775). Cognitive impairment was assessed per Langa-Weir’s classification, which includes normal, cognitively impaired but not demented (CIND) and demented groups. Generalized Estimating Equations (GEE) regressions estimated odds ratios (ORs) and 95% confidence intervals (CI) with adjustment for socio-demographic and lifestyle factors. Having at least one early learning problem, and death of a parent before the age of 16 were each associated with increased risk of later life cognitive impairment over time (OR 1.60, 95% CI 1.45-1.76 and OR 2.02, 95%CI 1.83-2.24 respectively). However, learning problem-related differences in risk of cognitive impairment varied by parental death status over time (learning problems x death x time, p=0.05). The risk was highest, amongst those that reported both experiencing early learning problems and parental death relative to those without learning problems. Policies and interventions that enhance diagnosis of early learning problems and improve childhood social contexts are needed to promote healthy cognitive aging amongst older Americans.

